# Editorial: Global advances in the diagnosis, management, and treatment of low back pain

**DOI:** 10.3389/fmed.2025.1554748

**Published:** 2025-02-07

**Authors:** Eron Manusov, Plamen Todorov, Vincent Diego

**Affiliations:** ^1^Department of Human Genetics School of Medicine, The University of Texas Rio Grande Valley, Brownsville, TX, United States; ^2^South Texas Diabetes and Obesity Institute, The University of Texas Rio Grande Valley, Brownsville, TX, United States; ^3^Department of Internal Disease Propaedeutics and Rheumatology, Medical University of Plovdiv, Clinic of Rheumatology, University Hospital “Kaspela”, Plovdiv, Bulgaria

**Keywords:** LBP, diagnosis, management, global advances, treatment

## Introduction

Low back pain (LBP) is a global health and quality of life concern, affecting millions of individuals and posing significant challenges to healthcare systems worldwide. This Research Topic, Global Advances in the Diagnosis, Management, and Treatment of Low Back Pain, includes a collection of studies addressing this prevalent condition's multifaceted nature. The Topic reviews various aspects of LBP, from diagnosis and treatment to its impact on specific populations and healthcare systems, highlighting innovative approaches and emerging trends across various healthcare levels. The articles include current reviews of epidemiology, diagnosis, treatment modalities, occupational health, patient perspectives, and healthcare system approaches to diagnosing and managing LBP. We aim to explore various diagnostic approaches, therapeutic interventions, and strategies for effectively managing LBP symptoms.

Low back pain (LBP) remains a significant global health concern, affecting up to 84% of people in their lifetime and leading to substantial disability and socioeconomic burden (Ferdinandov). Low back pain affects populations traditionally not considered at risk for LBP. There is a high prevalence of musculoskeletal pain, including LBP, among university students, with electronic device use and lack of exercise as key contributing factors (Kandasamy et al.). Almansour et al. reported a high prevalence of LBP (62.56%) among secondary school teachers in Saudi Arabia, with age, female gender, and increased workload reported as significant predictors. Hu et al. found that sleeping < 6.55 h per day was associated with a higher risk of LBP in adults over 50. The study by Ding et al. on emerging manufacturing workers in Beijing, China, further emphasizes the occupational aspect of LBP, reporting the highest prevalence in the neck (15.0%), followed by the lower back (12.5%) and shoulders (11.2%). Other occupations targeted for prevention are highlighted by Hakiranuye et al., who examined the prevalence of LBP among medical trainees and the implications for choosing future medical careers. Zhang et al. reviewed factors affecting functional disability, including lower educational background and posture. These findings highlight the significant impact of LBP on occupational health and career choices, emphasizing the need for workplace interventions and ergonomic considerations.

The diagnosis of LBP is difficult due to the multi-factorial etiology and varying presentation. Improving diagnostic accuracy for LBP remains a critical area of research. Ferdinandov et al. provide a narrative review of common differential diagnoses of LBP in contemporary medical practice, emphasizing the importance of a comprehensive diagnostic approach that considers intrinsic spinal, systemic, and referred pain sources. Morimoto et al. conducted a scoping review on gait analysis using digital biomarkers, including smart shoes, in lumbar spinal canal stenosis, highlighting the potential of wearable technology in LBP diagnosis. These studies emphasize the complexity of LBP diagnosis and the potential for innovative technologies to enhance diagnostic accuracy. The use of digital biomarkers and wearable technology provides for a more precise and objective assessment of LBP (Morimoto et al.).

Effective alternative treatment options for the management of LBP are reviewed. Ferdinandov conducted a systematic review on focused extracorporeal shockwave therapy (ESWT) for LBP treatment. Li W. et al. proposed a protocol for a systematic review and meta-analysis of the efficacy of silver needle therapy for treating LBP. Li X. et al. performed a meta-analysis on the clinical efficacy of acupuncture therapy combined with core muscle exercises in treating chronic non-specific LBP demonstrating favorable outcomes compared to single-core muscle training. These studies highlight the diverse treatment options for LBP and the ongoing efforts to evaluate their efficacy.

Advancements in surgical and anesthetic approaches are also included in this Topic. Mao-jiang et al. evaluated the efficacy and safety of CT-guided joint cavity release for postpartum sacroiliac joint pain management. Boykov et al. investigated thoracic spinal anesthesia with intrathecal sedation for lower back surgery, presenting a potential alternative to general anesthesia. Yankov et al. assessed multidetector CT Hounsfield unit measurements to predict efficacy and complications in percutaneous vertebroplasty for osteoporotic vertebral compression fractures. These studies demonstrate ongoing innovations in surgical and anesthetic techniques for LBP management, offering potential alternatives to traditional approaches and improving patient outcomes.

Liew and Darlow used network analysis to explore the complexity of commonly held attitudes and beliefs about LBP. Attitudes and beliefs affect not only diagnosis but also treatment and outcomes in a cross-sectional study. Mathieu et al. evaluated the appropriateness of specialized care referrals for LBP. There are a significant number of inappropriate referrals to neurosurgeons. Unnecessary surgical referrals increase costs and reduce patient and physician satisfaction. These studies underscore the importance of addressing patient beliefs and optimizing healthcare referral systems in LBP management. The high rate of inappropriate referrals highlights the need for improved triage and referral processes in primary care.

The Research Topic includes innovative approaches demonstrating the ongoing efforts to develop new strategies for LBP management and incorporating technology and coordinated care models to improve patient outcomes. García-López et al. present a pilot randomized controlled trial protocol for using virtual reality to improve low back and pelvic pain during pregnancy. Ramond-Roquin et al. proposed a protocol for coordinated care to reduce the risk of prolonged disability among patients with subacute or recurrent acute LBP in primary care.

## Conclusion

This Topic highlights the multifaceted nature of LBP research, encompassing epidemiology, diagnosis, treatment modalities, occupational health, patient perspectives, and healthcare system approaches. The studies provide information to improve the understanding and management of LBP. Future research should focus on implementing evidence-based strategies to improve patient outcomes and reduce the global burden of LBP. Particular attention should be given to:

1. Developing and validating innovative diagnostic tools, including digital biomarkers and wearable technology.
2. Evaluating the long-term efficacy of emerging treatment modalities such as ESWT and silver needle therapy.
3. Implementing and assessing the effectiveness of coordinated care models in primary care settings.
4. Addressing occupational factors and developing targeted interventions for high-risk professions.
5. Improving referral processes and triage systems to ensure appropriate utilization of specialized care.

By addressing these areas, we can work toward more effective, personalized, and efficient management of LBP, ultimately improving the quality of life for millions of individuals.

